# Silent but Distinct: Rare Adenoid Cystic Carcinoma of the Breast

**DOI:** 10.7759/cureus.111069

**Published:** 2026-06-18

**Authors:** Soumiya Samba, Soufiane Berhili, Ahmed BenSghier, Mohamed Moukhlissi, Loubna Mezouar

**Affiliations:** 1 Department of Radiation Oncology, Mohammed VI University Hospital, Faculty of Medicine and Pharmacy, Mohammed First University, Oujda, MAR

**Keywords:** adenoid, breast, carcinoma, cystic carcinoma, radiation

## Abstract

Adenoid cystic carcinoma (ACC) of the breast is an exceptionally rare malignancy, representing less than 0.1% of all breast cancers. Although it often demonstrates a triple-negative immunophenotype, its clinical course is typically more indolent than conventional triple-negative breast carcinomas. We report the case of a 64-year-old woman who presented with spontaneous nipple discharge from the left breast. Imaging revealed a suspicious lesion (Breast Imaging-Reporting and Data System (BI-RADS 5), leading to a core needle biopsy and tumorectomy, which identified a 2 cm invasive carcinoma of no special type. Completion mastectomy with axillary lymph node dissection revealed an 18 mm residual grade 1 tumor with clear margins and no nodal involvement. Immunohistochemistry confirmed a triple-negative profile, and staging CT scans showed no evidence of distant metastasis. The patient subsequently underwent adjuvant radiotherapy to the left chest wall.

Given these findings, including complete surgical resection, absence of nodal involvement, and lack of distant metastasis, the clinical course is consistent with the favorable prognosis typically observed in breast ACC. This case highlights the diagnostic challenges, distinctive pathological features, and favorable outcomes associated with this rare entity, emphasizing the importance of accurate histopathologic evaluation and appropriate surgical management in guiding long-term prognosis.

## Introduction

Adenoid cystic carcinoma (ACC) of the breast is a rare malignancy, accounting for fewer than 0.1% of all primary breast cancers [1,2]. It represents a distinct histologic subtype that closely resembles ACC of the salivary glands, although its clinical behavior in the breast is generally more indolent [3]. Histologically, breast ACC is composed of two cellular components, epithelial (luminal) and myoepithelial (basaloid) cells, arranged in characteristic architectural patterns. The cribriform pattern is most common, followed by tubular-trabecular and, less frequently, solid growth forms [4]. These features produce the classic "Swiss-cheese" appearance, also observed in salivary gland ACC, characterized by true glandular lumina and pseudo-lumina filled with basement membrane-like material.

Although ACC of the breast typically exhibits a triple-negative immunophenotype (absence of oestrogen receptor, progesterone receptor, and HER2 expression), it behaves far less aggressively than conventional triple-negative breast carcinomas [3]. Most cases are low-grade tumours with slow growth and limited metastatic potential. A recurrent chromosomal translocation, t(6;9), resulting in the MYB-NFIB gene fusion, is frequently identified and reflects its distinct molecular pathogenesis [5]. Clinically, ACC most often presents as a well-circumscribed breast mass, particularly in women over 40 years of age. It frequently arises in the subareolar region, although involvement of the overlying skin or nipple remains uncommon [6,7].

The diagnosis of breast ACC can be challenging, given its morphological overlap with several other entities. Key differential diagnoses include cribriform carcinoma in situ, collagenous spherulosis, and invasive cribriform carcinoma, as well as basaloid carcinomas and other rare salivary gland-type tumours of the breast [5-7]. Immunohistochemistry (IHC) plays a central role in establishing the correct diagnosis. The characteristic dual-cell population is highlighted by the co-expression of luminal markers (such as CK7 and CD117/c-Kit) and myoepithelial markers (including p63, smooth muscle actin, and calponin). CD117 expression, in particular, is a diagnostically useful marker that is consistently positive in ACC and helps distinguish it from its mimics [5-7]. The MYB-NFIB fusion, detectable by fluorescence in situ hybridization (FISH) or molecular testing, further supports the diagnosis in equivocal cases [5].

Given its rarity and unique clinicopathological profile, each newly reported case of breast ACC contributes valuable insight into this uncommon tumour entity.

## Case presentation

A 64-year-old married woman, mother of 3, was referred to our oncology department for continuation of care following surgical management of left breast cancer. She was managed at the Mohammed VI University Hospital, Oujda, Morocco. Her medical, surgical, familial, and toxicological histories were unremarkable.

The patient reported that her symptoms had begun approximately six months earlier, presenting as spontaneous nipple discharge from the left breast. She initially sought evaluation in the private sector, where she underwent bilateral breast ultrasonography and mammography, followed by a core needle biopsy of the left breast and subsequent tumorectomy. Staging investigations led to a left mastectomy with axillary lymph node dissection.

She was then referred to our institution for further oncologic management. After comprehensive assessment, adjuvant chemotherapy was deemed unnecessary based on the histopathological findings.

**Figure 1:**
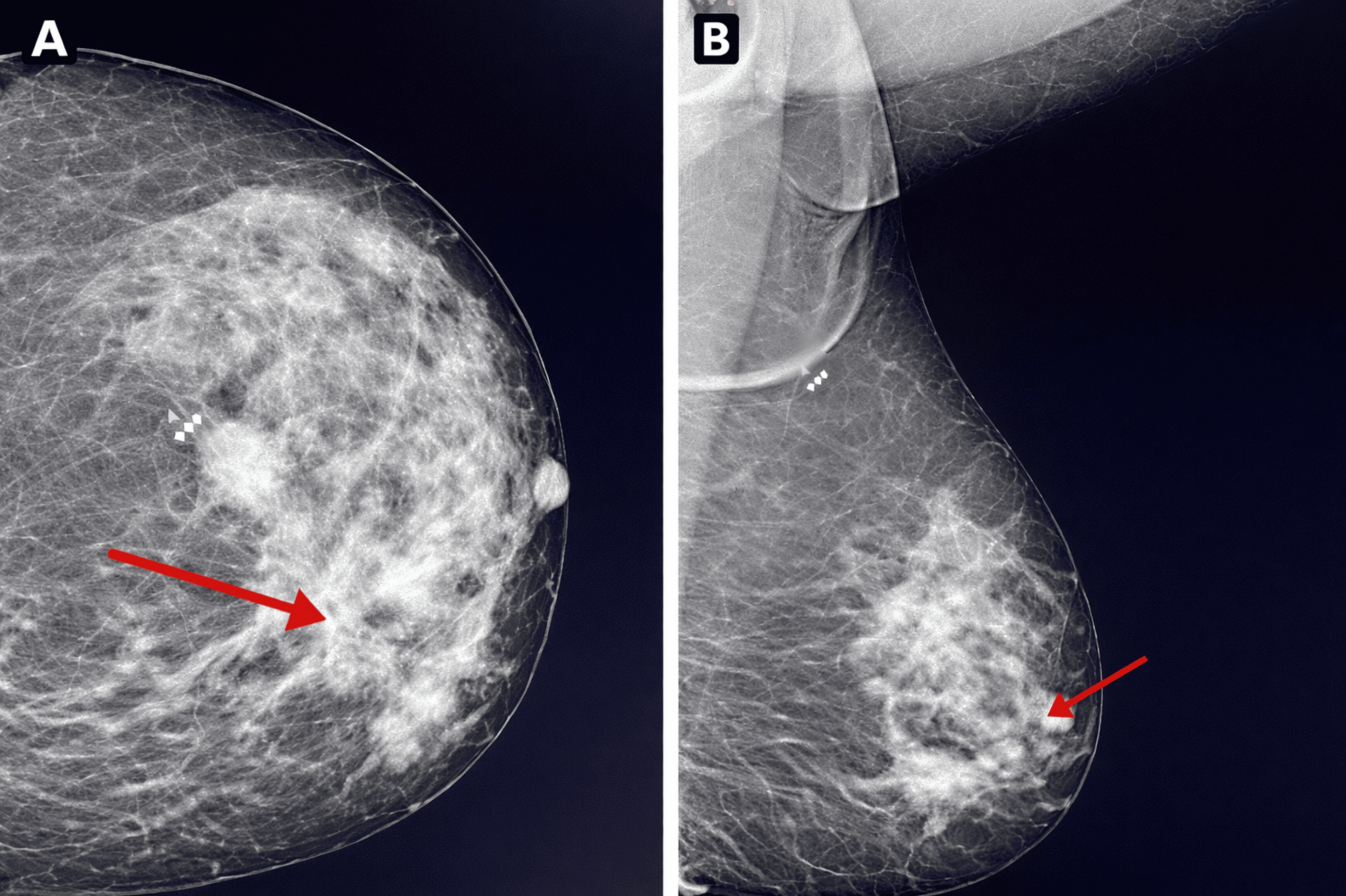
Mammography demonstrating a lesion at the junction of the inner breast quadrants in a 64-year-old patient (A) Craniocaudal (CC) view shows a focal suspicious lesion within the medial breast, located at the junction of the upper and lower inner quadrants (arrow). (B) Mediolateral oblique (MLO) view confirms the same finding in the corresponding region (arrow), demonstrating concordant localization across standard projections.

Bilateral breast ultrasonography and mammography identified a lesion in the upper inner quadrant (UIQ) of the right breast classified as BI-RADS (Breast Imaging-Reporting and Data System) 3, and a suspicious lesion in the UIQ of the left breast categorized as BI-RADS 5 (Figure 1).

Laboratory investigations, including complete blood count, renal function tests, liver enzymes, and inflammatory markers, were within normal limits.

Core needle biopsy of the left breast showed no definitive evidence of malignancy in the available tissue samples.

**Figure 2:**
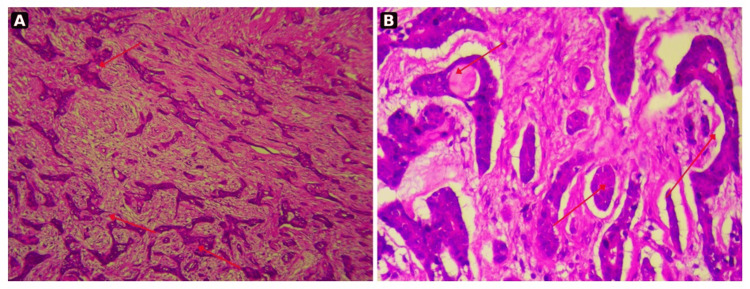
Histopathological examination of a breast tumor in a 64-year-old patient (A) Low-power view showing an infiltrative epithelial neoplasm composed of irregular nests and cords embedded within a fibrocollagenous stroma. The tumor demonstrates a characteristic cribriform and tubular architecture with pseudoglandular spaces. (B) High-power view highlighting classic features of adenoid cystic carcinoma, including pseudolumina and true lumina within tumor nests, as well as hyaline basement membrane-like material within pseudocystic spaces, reflecting dual epithelial and myoepithelial/basal cell differentiation.

Left breast tumorectomy revealed a 2 cm invasive carcinoma of no special type (NST) with a minor in situ component. No vascular emboli were identified, and the deep surgical margin measured less than 1 mm. Completion mastectomy with axillary lymph node dissection demonstrated an 18 mm residual grade 1 tumor with clear surgical margins. All 18 axillary lymph nodes were free of metastatic involvement (0/18), corresponding to a final pathological stage of pT1bN0M0 (Figure 2).

**Figure 3:**
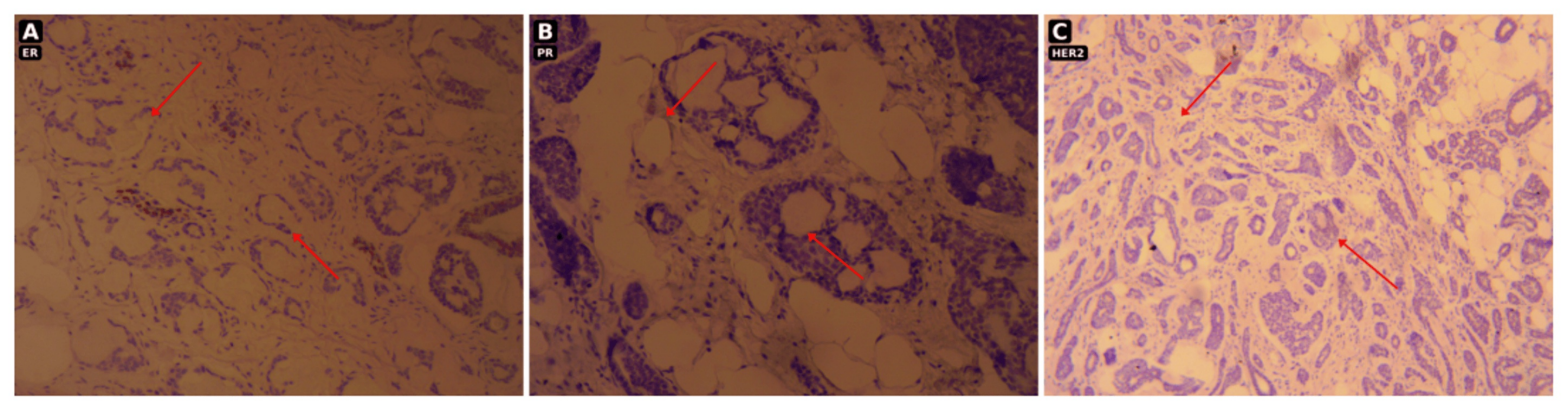
Immunohistochemical profile of breast adenoid cystic carcinoma in a 64-year-old patient (A) Estrogen receptor (ER): Tumor nests show absence of nuclear staining in neoplastic cells. (B) Progesterone receptor (PR): Tumor nests demonstrate no nuclear expression in tumor cells. (C) Human epidermal growth factor receptor 2 (HER2): Tumor nests show no membranous overexpression (non-amplified pattern), consistent with a typical adenoid cystic carcinoma immunophenotype.

Immunohistochemical analysis demonstrated CD117 (c-KIT) positivity in the epithelial tumor cells and P40 positivity in the basal/myoepithelial cells, confirming the characteristic biphasic cell population of ACC. The tumor exhibited a triple-negative phenotype (ER−, PR−, HER2−, score 0) (Figure 3).

**Figure 4:**
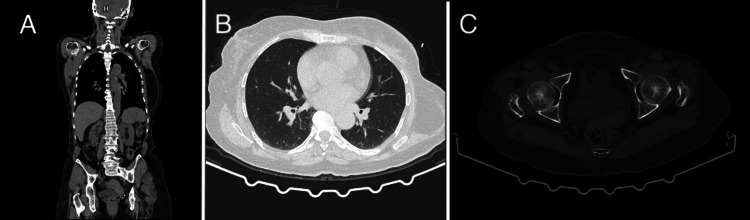
Thoraco-abdominopelvic computed tomography (CT) scan showing no evidence of distant metastatic disease in a 64-year-old patient (A) Coronal CT reconstruction demonstrating absence of detectable visceral, nodal, or osseous metastases. (B) Axial chest CT scan showing no evidence of pulmonary metastatic lesions. (C) Axial pelvic CT scan demonstrating no focal suspicious lesions.

A thoraco-abdominopelvic CT scan showed no evidence of distant metastatic disease (Figure 4).

Upon presentation to our department, the patient was alert and in good general condition. Physical examination showed a well-healed surgical scar on the left chest wall. The right breast appeared normal on inspection and palpation. No palpable axillary or supraclavicular lymphadenopathy was detected, and there were no signs of lymphedema or restricted shoulder mobility.

**Figure 5:**
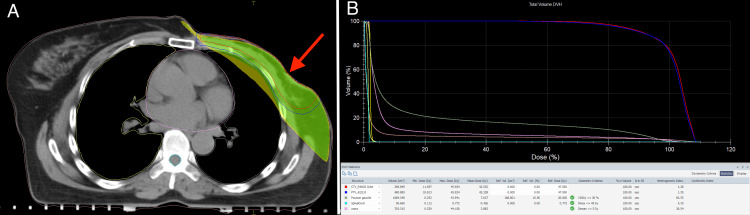
Adjuvant radiotherapy planning for the left chest wall in a 64-year-old patient (A) Axial planning computed tomography (CT) demonstrating target volume delineation and dose distribution for the left chest wall (arrow). (B) Corresponding dose–volume histogram (DVH) illustrating target coverage and organs-at-risk constraints. The prescribed dose was 42 Gy delivered in 15 fractions (2.8 Gy per fraction).

For adjuvant radiotherapy planning, a simulation CT scan was performed in a reproducible and comfortable position. The treatment plan consisted of delivering 42 Gy in 15 fractions (2.8 Gy per fraction) to the left chest wall (Figure 5).

## Discussion

Primary ACC of the breast is an exceptionally rare malignancy, representing fewer than 0.1% of all breast carcinomas [1,2]. In the United States, its age-adjusted incidence is estimated at approximately 0.92 cases per million person-years [3]. This tumor predominantly affects women in the fifth to sixth decades of life (median age ~60 years), although cases have been reported across a wide age range, from the third to the ninth decade. Occasional cases have also been documented in male patients [2]. Most patients present with a solitary, palpable breast mass, typically without skin changes or nipple discharge [6]. Pain or tenderness is uncommon and is thought to be related to perineural invasion [7].

Imaging features are nonspecific. On mammography and ultrasonography, ACC usually appears as an irregular, heterogeneous mass and is commonly classified as BI-RADS 4 [2]. Unlike invasive ductal carcinoma, microcalcifications are generally absent [2]. In some cases, ACC may mimic benign lesions such as fibroadenomas or phyllodes tumors [2,8]. Therefore, histopathological evaluation remains essential. Fine-needle aspiration or core needle biopsy may show basaloid cells and hyaline globules [6,9], but definitive diagnosis relies on histologic examination supported by immunohistochemistry [10].

This case illustrates the diagnostic complexity that can be encountered with breast ACC. Notably, our patient presented with nipple discharge, an atypical and rarely reported symptom of this entity, which initially oriented the clinical workup toward more common etiologies. Furthermore, the initial core needle biopsy failed to yield a definitive diagnosis, underscoring that a negative biopsy result should not lead to premature exclusion of malignancy when clinical suspicion remains high. On preliminary histological examination, the tumor also displayed morphological features overlapping with invasive carcinoma of no special type (NST), and the correct diagnosis was only established after thorough histopathologic re-evaluation supported by immunohistochemistry. This case therefore highlights that breast ACC should not be approached as a simple pattern-recognition exercise, but rather that clinicians and pathologists must maintain a high index of suspicion and pursue a comprehensive pathological workup when faced with atypical or diagnostically challenging breast lesions.

Histologically, ACC is characterized by a biphasic architecture composed of epithelial and myoepithelial cells arranged in cribriform and tubular patterns. Immunohistochemical analysis typically shows a triple-negative phenotype, with frequent expression of c-KIT (CD117), CK5/6, CK14, and p63 [4,10].

From a molecular perspective, breast ACC exhibits a distinct genetic profile compared with conventional triple-negative breast carcinomas. A recurrent chromosomal translocation t(6;9)(q22-23;p23-24) has been frequently identified, resulting in the MYB-NFIB gene fusion [10-12]. This fusion leads to overexpression of the MYB transcription factor, which plays a central role in tumorigenesis through regulation of cell proliferation, differentiation, and inhibition of apoptosis. This molecular hallmark is considered a defining feature of ACC and is closely associated with its characteristic biphasic morphology and relatively indolent clinical behavior despite its triple-negative immunophenotype.

The differential diagnosis includes collagenous spherulosis (a benign mimic), invasive cribriform carcinoma (typically ER-positive), metaplastic carcinoma, and basal-like carcinomas [9,10]. The distinction is based on the recognition of the biphasic pattern and immunohistochemical profile. Rarely, metastatic ACC from salivary gland origin must also be excluded [2].

Given the rarity of breast ACC, standardized treatment guidelines are not well-established [2]. Surgical excision remains the cornerstone of management. Although mastectomy was historically the preferred approach, increasing evidence supports breast-conserving surgery followed by adjuvant radiotherapy as an effective strategy with excellent local control [11]. Comparable local recurrence rates have been reported between both approaches [11].

Axillary lymph node involvement is rare, occurring in fewer than 2% of cases; therefore, sentinel lymph node biopsy is generally sufficient [2,11]. Axillary dissection is reserved for clinically or histologically positive nodes [11].

Adjuvant chemotherapy is rarely indicated due to limited benefit. It may be considered in selected high-risk cases such as tumors larger than 3 cm, nodal involvement, or solid histological variants [12]. Hormonal therapy is generally not effective, as most tumors are triple-negative; however, ER-positive cases may be managed individually [10].

Overall, breast ACC has an excellent prognosis, with reported 5-year survival rates exceeding 95% [1-3]. Local recurrence occurs in approximately 3-18% of cases and is usually amenable to surgical resection [2]. Distant metastases are rare and most commonly involve the lungs, followed by the liver, bones, and kidneys [2].

Long-term follow-up is recommended due to the possibility of late recurrence [11,12]. Prognosis is influenced by tumor size and histological grade, with high-grade (solid variant) tumors associated with more aggressive behavior [12]. However, most cases are low- or intermediate-grade and demonstrate durable long-term survival [3].

This report has several limitations. It describes a single patient, which limits the generalizability of the findings. In addition, the follow-up period remains relatively short, preventing definitive conclusions regarding long-term recurrence risk and survival outcomes. Therefore, the results should be interpreted with caution, and larger studies with longer follow-up are required to better characterize the clinical behavior and optimal management of breast adenoid cystic carcinoma.

## Conclusions

Primary ACC of the breast is a rare and distinct tumor characterized by a biphasic histologic pattern, triple-negative phenotype, and overall favorable prognosis. Despite nonspecific clinical and radiologic features, accurate diagnosis relies on careful histopathologic and immunohistochemical evaluation. Surgical excision remains the mainstay of treatment, with breast-conserving surgery followed by radiotherapy increasingly recognized as an effective option for local control. Lymph node involvement and distant metastasis are uncommon, reinforcing the generally indolent nature of this disease. Long-term surveillance, however, remains essential due to the possibility of late recurrence. This case underscores the importance of recognizing this uncommon entity and contributes to the growing body of literature guiding evidence-based management of breast ACC.
